# Comparison of Quality Characteristics of Commercial Kimchi Manufactured in Korea, China, and the United States

**DOI:** 10.3390/foods10102488

**Published:** 2021-10-18

**Authors:** Ye-Rang Yun, Jin Ju Lee, Hye Jin Lee, Yun-Jeong Choi, Jong-Hee Lee, Sung Jin Park, Sung Hee Park, Hye-Young Seo, Sung Gi Min

**Affiliations:** 1 Research and Development Division, World Institute of Kimchi, Nam-Gu, Gwangju 61755, Korea; yunyerang@wikim.re.kr (Y.-R.Y.); hjl0617@wikim.re.kr (H.J.L.); yjchoi85@wikim.re.kr (Y.-J.C.); leejonghee@wikim.re.kr (J.-H.L.); parksungjin@wikim.re.kr (S.J.P.); shpark@wikim.re.kr (S.H.P.); hyseo@wikim.re.kr (H.-Y.S.); 2 Pulmuone Institute of Technology, Cheongju 28164, Korea; jjleen@pulmuone.com

**Keywords:** kimchi, free sugar, organic acid, free amino acid, volatile compound, microbial community, antioxidant activity

## Abstract

Recently, kimchi has been recognized as a healthy food worldwide, prompting increased interest in its health benefits and quality characteristics. Although commercial kimchi is manufactured in various countries, little is known about quality differences between the kimchi from different countries. To clarify differences in quality characteristics, minerals, free sugars, organic acids, free amino acids, and volatile compounds, commercial kimchi manufactured in Korea, China, and the United States were investigated. The composition of the microbial community and antioxidant activity were compared. Mineral and free sugar contents were high in Korean commercial kimchi, while the organic acid content was relatively low. The free amino acid content was markedly higher in Korean kimchi than that in kimchi manufactured in China and the United States. In addition, the volatile compound content differed between the kimchi produced in different countries. Considering the microbial communities, *Leuconostoc* and *Weissella* were more abundant in commercial kimchi from Korea than that from China or the United States. Commercial kimchi in Korea showed the highest antioxidant activity. These results support the high quality and antioxidant activity of commercial kimchi manufactured in Korea, emphasizing its importance in the global kimchi industry.

## 1. Introduction

Kimchi is a well-known traditional Korean fermented food. In 2001, it was officially recognized as CODEX standard [1], and kimchi cabbage was listed in CODEX standard as a synonym for Chinese cabbage and napa cabbage in 2012. Moreover, kimjang (making and sharing kimchi) was proudly listed in UNESCO’s list of the intangible cultural heritage of humanity in 2013 [2]. Kimchi is commonly known to contain abundant nutrients and functional ingredients; and therefore, it exhibits various health benefits, including antioxidant [3,4,5], anti-obesity [6,7,8], anti-diabetic [9,10], and anti-cancer effects [11,12], among others.

Fermented cabbage inhibits the production of angiotensin-converting enzyme 2 (ACE 2), which is the binding site for coronavirus, aiding in its crossing of the cell membrane and therefore preventing COVID-19 [13,14]. Accordingly, interest in its health benefits is increasing worldwide, along with expectations for vegetable fermented food, including kimchi, sauerkraut, and suancai for the prevention of COVID-19. There is ongoing kimchi research focused on its health benefits. Kimchi supplemented with Jeju citrus concentrate shows anti-obesity effects in vitro and in vivo by reducing triglyceride levels and downregulating the expression of obesity-related genes [7,8]. In addition, the functionality of lactic acid bacteria (LAB) isolated from kimchi is analyzed extensively [15,16,17]. *Lactobacillus fermentum* SMFM2017-NK4 isolated from kimchi exhibits an anti-obesity effect in mice by inhibiting fat accumulation [16]. In addition to the research on the health functionality of kimchi, research on kimchi quality is important. However, there are only a few studies examining the quality of kimchi and comparing the quality of kimchi originating from different countries.

The demand for kimchi is increasing; therefore, it is either manufactured and exported from Korea or manufactured and sold in other countries [18]. In particular, the production and consumption of kimchi is high in China, Japan, and the United States. In addition, kimchi exports to Hong Kong, Taiwan and Australia are increasing. However, kimchi research is mainly conducted in Korea and has largely focused on an analyses of quality based on fermentation properties [19,20,21]. To date, there has been no report analyzing and comparing the quality characteristics of kimchi produced in different countries. Additionally, the relationship between the origin of kimchi and its quality remains controversial. Interestingly, Lee et al. identified 23 proteins that were differentially expressed between Korean and Chinese kimchi, using proteomic analysis, suggesting a difference in kimchi properties based on geographic origin [22]. Therefore, a comparison of the quality of kimchi originating from different countries is required. 

In this study, we compared commercial kimchi manufactured in Korea, China, and the United States for their mineral composition, free sugar, organic acid, free amino acid, and volatile compound contents. We determined their bacterial community composition. In addition, antioxidant activity levels were investigated.

## 2. Materials and Methods

### 2.1. Materials

All reagents were obtained from Sigma Aldrich (St. Louis, MO, USA). A total antioxidant capacity (TAC) assay kit was also obtained from Sigma Aldrich (MAK187). Ferric reducing antioxidant power (FRAP) colorimetric detection kit was obtained from Arbor Assays (K043-H1, Ahn Arbor, MI, USA).

### 2.2. Commercial Kimchi Sample Collection

To compare the quality characteristics, the United States, China, and Japan were initially considered as suitable sources, based on the import and export status of kimchi and the local market. However, Japan was excluded owing to a local situation. Information on commercial kimchi collected from Korea, China, and the United States is presented in Supplementary Table S1.

In Korea, China, and the United States, 5–6 pieces of each commercial kimchi product were selected based on the volume and location of the producer, because we wanted to ensure the reliability of the sample by choosing producers from various locations rather than focusing on a specific region. There are numerous kimchi producers in Korea, China, and the United States; however, 5–6 companies in each country occupy more than 60% of the kimchi market share. We agree that the number of samples used in study is small; however, producers in various locations with a high market share in each country were selected. We believe that this is sufficiently representative. We selected producers from Oregon, Santa Rosa, LA, and NY in the US; from Beijing, Shanghai, and Qingdao in China’ and from Gangwon-do, Chungcheong-do, and Jeolla-do in Korea. Korean traditional kimchi, which is not sterilized and is naturally fermented, was directly purchased from a local store with a similar manufacturing date and used as an analysis sample. The most common kimchi is fusion-style kimchi that combines locally produced vegetables and red pepper sauce with the traditional Korean kimchi fermentation method. The samples from different countries varied with respect to the packaging type and ingredients. In the United States, most commercial kimchi products are packaged in 300–400 g glass bottles for the local market, whereas 1–1.4 kg plastic containers are used for Korean or large discount stores. Few ingredients in addition to the kimchi cabbage and seasoning are used; and special ingredients, additives, and fish sauce are rarely used. Commercial kimchi in Korea and China shows similar ingredients and packaging characteristics; however, additives (such as potassium sorbate, colorant, sodium l-glutamate (including nucleic acids), alcohol, and sodium dehydroacetate) were more frequently used in China than in Korea. In Korea, kimchi cabbage, seasoning, various fish sauces, and umami ingredients are commonly used, in addition to LAB starters including *Leuconostoc mesenteroide* and *Leuconostoc* *citreum*. Commercial kimchi samples manufactured in Korea, China, and the United States were used in the experiment when pH reached a ripening point of pH 3.8−4.1. Each kimchi sample manufactured in Korea, China, and the United States was homogenated. Data are expressed as mean ± standard deviation (SD) for each country.

### 2.3. Salinity and Capsaicinoid Contents Analysis

Kimchi juice was prepared by blending all of the kimchi samples. Kimchi juice was diluted with 0.9% saline solution (HAPS DW-9, HUKO FS Co., Ltd., Seoul, Korea) and filtered. Initially, 1 mL of 2% potassium chromate was added and titrated against 0.02 N AgNO_3_ until reaching a red-brown color. The capsaicinoid content of kimchi was analyzed using high-performance liquid chromatography (HPLC). The sample was diluted, filtered with filter paper, and evaluated using an HPLC analyzer (Agilent Technologies, Santa Clara, CA, USA) coupled with a fluorescence detector (Agilent Technologies). Excitation and emission wavelengths were 208 and 325 nm, respectively. Capsaicinoid contents were calculated from the peak sizes for capsaicin and dihydrocapsaicin chromatograms.

### 2.4. Mineral Contents Analysis

Mineral contents were examined. Kimchi samples were placed in a furnace at 600 °C for 12 h and subsequently cooled. Samples were reacted with 6 M HCl for 15 h and filtered. Then, 100 μL of the mixture was diluted with 3 mL distilled water and fed into inductively coupled plasma mass-spectrophotometry (ICP-MS, Model # 7500a; Agilent Technology, Palo Alto, CA, USA) to measure the mineral composition.

### 2.5. Free Sugar Contents Analysis

The free sugar contents of kimchi were measured using HPLC. The sample was diluted, filtered with 0.22 μm filter paper, and evaluated using an HPLC analyzer (Dionex Ultimate3000 attached to Sugar-pak for free sugar; Thermo Dionex, Waltham, MA, USA). To assess free sugars, the mobile phase was eluted with a 100% H_2_O solution (temperature 70 °C, flow rate 0.5 mL/min, Shodex RI-101 detector, 210 nm, and injection volume 10 μL). Free sugar contents were calculated from the chromatogram.

### 2.6. Organic Acid Contents Analysis

Organic acid contents in kimchi samples were measured using HPLC. The blended kimchi was diluted, filtered with 0.22 μm filter paper, and evaluated using an HPLC analyzer (Aminex 87H for organic acid; Thermo Dionex, Waltham, MA, USA). The mobile phase was eluted with 0.01 N H_2_SO_4_ solution (flow rate 0.5 mL/min, RI detector, 210 nm, and injection volume 10 μL). Organic acid contents were calculated from the chromatogram.

### 2.7. Free Amino Acid Contents Analysis

Kimchi samples were diluted, homogenized, and refrigerated for 15 h. After filtration with a 0.45 µm membrane filter, the sample solution was analyzed using HPLC (Ultimate 3000; Thermo Dionex) equipped with a VDSpher 100 C18-E column (4.6 × 150 mm, 3.5 µm; VDS optilab, Berlin, Germany) and a Detector (FL Detector 1260 FLD) in an oven at 40 °C. The content of each free amino acid was determined based on the values for the amino acid standards (Agilent Technologies).

### 2.8. Volatile Compounds Analysis

Volatile compound analysis was performed as described previously [23]. Volatile compounds were analyzed using gas chromatography–mass spectrometer (GC-MS 7890A; Agilent Technologies) equipped with a DB-WAX column (60 m × 0.25 mm × 0.25 µM) and an autosampler (Multi-Purpose Sample with DHS option, MPS, Gerstel, Germany); the samples were initially extracted using solid-phase microextraction (SPME) fibers (DVB/CAR/PDMS, 50/30 µM, Supelco-57329-U), and absorbed onto polydimethylsiloxane (PDMS) fibers at 100 rpm for 30 min at 50 °C. The SPME fibers (DVB/CAR/PDMS, 50/30 µM, Supelco-57329-U), which extracted the volatile compounds, were automatically injected into a GC-MS injection port, and the volatile compounds were thermally desorbed at 250 °C for 3 min and analyzed using GC-MS with a constant helium flow rate of 1 mL/min. The temperature programs were as follows: 40 °C for 3 min, 2 °C/min up to 150 °C, 150 °C for 10 min, 4 °C/min up to 200 °C, and a final hold at 200 °C for 10 min. Electron impact ionization (70 eV) was performed at a full scan range of 50–550 m/z. Each volatile compound was identified based on a mass spectral library (WILEY 10N). The amounts of the identified volatile compounds were measured from the peak areas of the GC/MS chromatograms for each sample, using the internal standard method and presented as μg/g.

### 2.9. Microbial Community Analysis

Microbial community analysis was performed according to the method proposed by a previous study [24]. Extracted total DNA from the kimchi samples was subjected to a PCR cycle using primers specific for the 16S V4 gene. Sequencing was conducted using the Mi-Seq™ platform (Illumina, San Diego, CA, USA) by Macrogen (Macrogen Inc., Seoul, Korea). After eliminating sequencing errors, as well as ambiguous and chimeric sequences, CD-HIT-OTU (operational taxonomic unit) was used to calculate the species-level OTUs using a similarity threshold of 97%. Additionally, representative sequences of each OTU were analyzed using UCLUST (v.1.2.22) in the reference database (SIVA DB) to generate taxonomic assignments based on homology. Microbial communities were analyzed using Ribosomal Database Project (RDP) classifiers in QIIME (v.1.8.0).

### 2.10. Antioxidant Activity Analysis

To analyze the antioxidant activity of all samples, the total phenol content (TPC), total flavonoid content (TFC), DPPH radical scavenging activity, TAC, and FRAP were measured. TPC was measured using the Folin-Ciocalteu method at 700 nm with a standard curve of gallic acid [25]. TFC was measured at 415 nm with a standard curve of quercetin as described by Chang et al. [26] DPPH radical scavenging activity was measured at 515 nm following the method described by Blois [27]. TAC and FRAP were measured using a commercial kit at 570 nm and 560 nm with a standard curve of Trolox. Fe (II), respectively.

### 2.11. Statistical Analysis

Data are presented as mean ± SD. Statistical significance was analyzed by one-way analysis of variance followed by Duncan’s multiple range test using GraphPad Prism 7 (GraphPad, Inc., San Diego, CA, USA). *p* < 0.05 was considered statistically significant.

## 3. Results and Discussion

### 3.1. Salinity and Casaicinoid Contents of Commercial Kimchi Manufactured in Korea, China, and the United States

The salinity levels of commercial kimchi from Korea and China were similar, while that of commercial kimchi from the United States was relatively high at 2.31 ± 0.41% (Table 1); however, the differences between the kimchi from the three countries was not significant. The capsaicinoid content was highest in samples from China (6.15 ± 4.14 ppm) and lowest in those from Korea (3.75 ± 2.22 ppm); however, the differences were not significant. According to the current KS standard of Korea, kimchi in Korea, China, and the United States can be classified as medium–hot based on the capsaicinoid contents [28]. In an analysis of 13 spicy commercial kimchi types, the capsaicinoid content ranged from 9.0–30.5 ppm and was classified as medium hot and hot [29]. Although there were no statistically significant differences in salinity and capsaicinoid content among the kimchi from the three countries, the standard deviation was the lowest in Korea, indicating low variation in quality among the Korean commercial kimchi samples, making them reliable.

### 3.2. Mineral Contents of Commercial Kimchi Manufactured in Korea, China, and the United States

The K, Mg, Ca, P, S, and Zn contents were substantially higher in Korean commercial kimchi than that in kimchi from China and the United States (Table 2). In particular, the K, Mg, P, S, and Zn contents were significantly higher in Korean samples than that in other samples (*p* < 0.05). The Na (9262 ± 1542 mg/kg) and Fe (0.55 ± 0.14 mg/kg) contents were significantly higher in samples from the United States compared with that from Korea and China (*p* < 0.05). Mineral contents in commercial kimchi from China were relatively low. In general, kimchi has a high mineral content, and Ca and P are abundant in most ingredients, especially in red pepper powder and *jeotgal* (salted shrimp, salted anchovies). The Na, K, Ca, and Mg contents were high in most kimchi, with some variation between samples in a previous study [30]. Our results suggest that the difference in mineral content could be attributed to the different ingredients used by different countries in manufacturing kimchi. These results indicate that kimchi manufactured in Korea is rich in minerals, which could have a beneficial effect on kimchi quality.

### 3.3. Free Sugar Contents of Commercial Kimchi Manufactured in Korea, China, and the United States

The levels of most free sugars were significantly higher in Korean commercial kimchi than that in other samples (Table 3, *p* < 0.05), except for that of mannitol. The sorbitol content of Korean commercial kimchi was substantially higher than that of commercial kimchi from China and the United States. The mannitol content was the highest in commercial kimchi from China (13,100 ± 2082 mg/kg). In kimchi, sugar is generally derived from the ingredients and it functions as a nutrient source for various microorganisms, giving kimchi its unique taste and flavor [31,32]. In this process, mannitol and sorbitol (sugar alcohol) are produced as metabolites of sugar-based microorganisms [33]. Therefore, this difference in free sugar could reflect the differences in sub-ingredients, such as garlic, red pepper powder, and glutinous rice paste, which are sugar sources in commercial kimchi samples. Additionally, these results indicate that microorganismal activity in Korean commercial kimchi is possibly high, conferring a positive effect on kimchi fermentation.

### 3.4. Organic Acid Contents of Commercial Kimchi Manufactured in Korea, China, and the United States

Organic acid contents were investigated using HPLC. Lactic acid, acetic acid, and ethanol contents were significantly higher in commercial kimchi from China than that in the other samples (Table 4, *p* < 0.05). Citric and fumaric acids were only detected in samples from the United States and Korea, respectively. Malic acid content decreases during the storage period, along with that of citric and fumaric acid; however, the lactic and acetic acid contents gradually increase during the kimchi fermentation process [34]. In addition, the organic acid in kimchi differs with the type of LAB and the degree of fermentation [24,35]. *Lactobacillus sakei* produce more lactic and acetic acids than *Lactobacillus mesenterioides* [23]. Based on these results, the difference in the organic acid content could be attributed to the differences in the microbial composition and fermentation properties.

### 3.5. Free Amino Acid Contents of Commercial Kimchi Manufactured in Korea, China, and the United States

Amino acids are produced or converted into metabolites by microorganisms [36]. Table 5 shows the free amino acid content of kimchi manufactured in Korea, China, and the United States. Among the free amino acids, glutamic acid, alanine, and glycine contribute to umami (savory taste), while leucine, isoleucine, and arginine contribute to bitterness [32]. Glutamic acid, alanine, and glycine contents in Korean commercial kimchi were significantly higher than that in kimchi from China and the United States (*p* < 0.05). In particular, the glutamic acid content was the highest in Korean commercial kimchi (1777 ± 342 mg/kg), compared to the relatively low levels in kimchi from China and the United States (308 ± 376 mg/kg and 357 ± 176 mg/kg, respectively). In contrast, the leucine and isoleucine contents were the highest in commercial kimchi from China. The arginine content was the highest in Korean commercial kimchi. The citrulline, ornithine, and 2-hydroxyisoc aproic acid (HICA) contents were the highest in Korean commercial kimchi. HICA is produced by LAB mainly during the early stage of fermentation, based on the LAB composition [37]. The difference in HICA contents indicates that the LAB composition differed among the samples from the different regions. Surprisingly, the highest levels of taurine and gamma-aminobutyric acid (GABA) were observed in the commercial kimchi from China. These differences could be attributed to the use of *jeotgal* (salted seafood) as a sub-ingredient. Korean commercial kimchi is relatively of high quality due to the high free amino acid content, which is closely related to umami.

### 3.6. Volatile Compounds Contents of Commercial Kimchi Manufactured in Korea, China, and the United States

Volatile compound contents were measured using GC-MS. Table 6 shows 36 volatile compounds detected in kimchi manufactured in Korea, China, and the United States. Major volatile compounds in Korean commercial kimchi were ethanol, β-phenethyl acetate, and benzenepropanenitrile, while major volatile compounds in commercial kimchi from China and the United States were ethanol, benzenepropanenitrile, and acetic acid. In our study, β-phenethyl acetate was detected at a high level in Korean commercial kimchi. β-Phenethyl acetate confers rose, honey, and sweet flavors and is used as a flavoring agent [38,39]. Additionally, 5-cyano-1-pentene, benzeneethanol, diallyl disulfide, methyl disulfide, and β-phenethyl_isothiocyanate were detected at high levels of 1 μg/g. These volatile compounds are produced from sulfur compounds in ingredients such as garlic and green onions. 1-methoxyhexane was absent from Korean commercial kimchi. 1-methoxyhexane imparts an ethereal, herbal, and fruity taste and it is derived from litchi (*Litchi chinesis* Sonn.) and *Salvia* species in food products [40]. The levels of diallyl disulfide, methanethiol, methyl disulfide, and octanoic acid were high in Korean commercial kimchi, reflecting the abundance of sulfur compounds from ingredients such as garlic and green onions. Therefore, the volatile compounds in different kimchi manufactured in Korea are similar; however, there are differences between the kimchi from different countries due to the differences in the sub-ingredients, such as garlic and green onion.

### 3.7. Microbial Community of Commercial Kimchi Manufactured in Korea, China, and the United States

**Figure 1:**
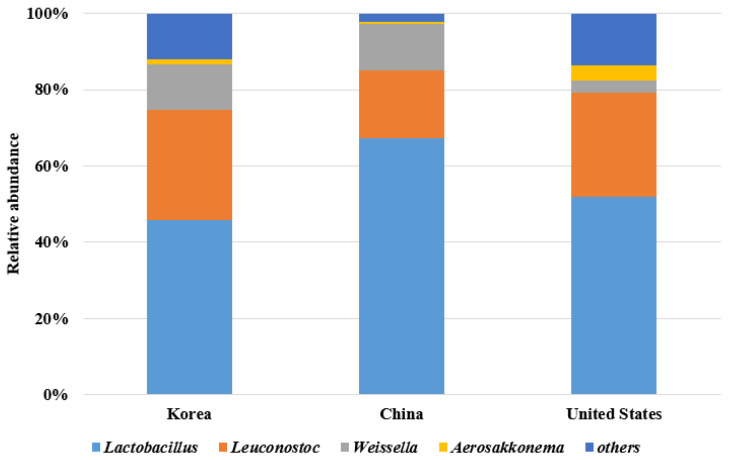
Microbial community of commercial kimchi manufactured in Korea, China, and the United States.

*Leuconostoc* is a dominant microbial community during the early kimchi fermentation stage, which creates an acidic environment under anaerobic conditions [41]. *Lactobacillus* rapidly increases under anaerobic conditions during the late stage [42,43]. Moreover, the microorganisms in kimchi show various growth patterns depending on the ingredients and the manufacturing environment [44,45]. The dominant fermentative microorganisms belong to the genera *Leuconostoc*, *Weissella*, and *Lactobacillus* in kimchi fermented by unsterilized ingredients, garlic, and kimchi cabbage, respectively [45]. The microbial communities in kimchi samples are presented in Figure 1. Korean commercial kimchi had the most operational taxonomic unit (OTUs, 2528.6, on average), indicating that a wide variety of microorganisms exist. In a genus-level analysis, *Lactobacillus* and *Leuconostoc* were abundant in samples from all countries. In samples from China, these genera were followed by *Weissella*; this was different to the microorganisms detected in Korean and American commercial kimchi. The relative abundance of *Lactobacillus* was extremely high (approximately 70%) in Chinese commercial kimchi, while *Weissella* was more abundant in the samples from Korea and China than that in the samples from the United States. These results indicate that Korean commercial kimchi contains diverse organisms, which could influence the fermentation and quality of the kimchi.

### 3.8. Antioxidant Activity in Commercial Kimchi Manufactured in Korea, China, and the United States

Antioxidant activity in commercial kimchi manufactured in Korea, China, and the United States is presented in Table 7. For all parameters related to antioxidant activity, values were higher for Korean commercial kimchi than that in the kimchi from China and the United States. TFC was higher for samples from Korea than those from China and the United States; however, the differences were not significant. The TPC, DPPH radical scavenging activity, TAC, and FRAP were significantly higher in Korean commercial kimchi compared with that in the other samples (*p* < 0.05). FRAP in Korean commercial kimchi was approximately 39% higher than that in Chinese commercial kimchi (1956.56 and 1398.95 μM, respectively). In previous studies, most kimchi showed high antioxidant activity, which is deemed to be caused by a physiologically active substance in the ingredients [3,7]. Therefore, the observed difference in the antioxidant activities could be attributed to the differences in the physiological properties of the ingredients. These results support the higher antioxidant properties and the wide microorganism composition of Korean kimchi.

## 4. Conclusions

We evaluated commercial kimchi manufactured in Korea, China, and the United States for a comprehensive comparison of quality characteristics. Korean commercial kimchi had a relatively low salinity and capsaicinoid content, high mineral and free sugar contents, a low organic acid content, and an extremely high free amino acid content. In addition, the volatile compounds and microbial community composition of Korean commercial kimchi differed to that of the kimchi from China and the United States. Furthermore, Korean commercial kimchi had the highest levels of antioxidant activity. Consequently, commercial kimchi manufactured in Korea shows high-quality characteristics and antioxidant activity.

## Figures and Tables

**Table 1:** Salinity and capsaicinoid contents of commercial kimchi manufactured in Korea, China, and the United States.

	Korea	China	United States
Salinity (%)	2.00 ± 0.15 ^NS^	2.06 ± 0.29	2.31 ± 0.4
Capsaicinoid (ppm)	3.75 ± 2.22 ^NS^	6.15 ± 4.14	5.11 ± 5.31

Data are expressed as the mean ± SD. ^NS^; Not significant differences among groups.

**Table 2:** Mineral contents of commercial kimchi manufactured in Korea, China, and the United States. (mg/kg).

	Korea	China	United States
Na	9039 ± 911 ^b^	8023 ± 765 ^b^	9262 ± 1542 ^a^
K	3677 ± 263 ^a^	2769 ± 384 ^b^	3082 ± 485 ^b^
Mg	271.3 ± 57.2 ^a^	214.9 ± 48.9 ^b^	226.6 ± 44.3 ^ab^
Ca	355.8 ± 40.1 ^NS^	341.7 ± 52.0	288.4 ± 87.6
Fe	0.42 ± 0.04 ^b^	0.48 ± 0.08 ^ab^	0.55 ± 0.14 ^a^
P	57.0 ± 5.8 ^a^	49.5 ± 3.8 ^ab^	44.4 ± 11.23 ^b^
S	47.2 ± 4.9 ^a^	40.9 ± 7.7 ^ab^	38.0 ± 7.2 ^b^
Zn	0.52 ± 0.04 ^a^	0.36 ± 0.05 ^b^	0.43 ± 0.10 ^b^

Data are expressed as the mean ± SD. Lowercase letters indicate significant differences among groups (*p* < 0.05). ^NS^; Not significant differences between groups.

**Table 3:** Free sugar contents of commercial kimchi manufactured in Korea, China, and the United States (mg/kg).

	Korea	China	United States
Sucrose	1031 ± 1197 ^a^	216 ± 241 ^b^	174 ± 211 ^b^
Glucose	10,239 ± 4934 ^NS^	5533 ± 4108	5962 ± 6321
Fructose	6733 ± 6148 ^a^	2916 ± 4333 ^ab^	1684 ± 3001 ^b^
Galactose	280 ± 88 ^a^	39 ± 57 ^b^	71 ± 107 ^b^
Mannitol	9329 ± 3398 ^b^	13,100 ± 2082 ^a^	12,158 ± 3879 ^ab^
Sorbitol	3375 ± 3454 ^a^	491 ± 862 ^b^	67 ± 25 ^b^

Data are expressed as the mean ± SD. Lowercase letters indicate significant differences among groups (*p* < 0.05). ^NS^; Not significant differences among groups.

**Table 4:** Organic acid contents of commercial kimchi manufactured in Korea, China, and the United States (mg/kg).

	Korea	China	United States
Lactic acid	7215 ± 1408 ^c^	15,266 ± 1655 ^a^	11,820 ± 4693 ^b^
Acetic acid	2998 ± 677 ^b^	4330 ± 793 ^a^	3690 ± 649 ^ab^
Citric acid	-	-	43.1 ± 71.9
Fumaric acid	16.33 ± 15.76	-	-
Et-OH	2629 ± 1449 ^b^	7750 ± 4009 ^a^	4101 ± 2440 ^b^

Data are expressed as the mean ± SD. Lowercase letters indicate significant differences among groups (*p* < 0.05).

**Table 5:** Free amino acid contents of commercial kimchi manufactured in Korea, China, and the United States (mg/kg).

	Korea	China	United States
Aspartic acid	332.9 ± 74.3 ^a^	114.5 ± 76.6 ^b^	120.5 ± 74.8 ^b^
Glutamic acid	1777 ± 342 ^a^	308 ± 376 ^b^	357 ± 176 ^b^
Asparagine	552.9 ± 80.1 ^NS^	563.2 ± 188.3	488.8 ± 149.9
Serine	394.2 ± 55.0 ^a^	168.0 ± 74.6 ^b^	135.6 ± 106.2 ^b^
Glutamine	2054 ± 180 ^a^	1494 ± 322 ^ab^	1399 ± 774 ^b^
Histidine	215.1 ± 159.0 ^b^	154.3 ± 86.5 ^b^	464.4 ± 143.9 ^a^
Glycine	280.4 ± 117.2 ^a^	217.3 ± 84.2 ^a^	123.4 ± 46.6 ^b^
Threonine	281.5 ± 35.6 ^a^	186.7 ± 92.0 ^b^	190.7 ± 49.0 ^b^
Citrulline	55.0 ± 16.2 ^a^	46.0 ± 16.2 ^a^	15.6 ± 9.9 ^b^
Arginine	454.0 ± 325.0 ^a^	16.4 ± 9.3 ^b^	150.0 ± 197.1 ^b^
Alanine	1003.7 ± 141.8 ^a^	1042.0 ± 520.0 ^a^	648.5 ± 243.0 ^b^
Taurine	26.5 ± 7.7 ^a^	32.5 ± 15.7 ^a^	2.8 ± 4.7 ^b^
GABA	433.1 ± 114.7 ^b^	1406.0 ± 1014.0 ^a^	436.5 ± 69.3 ^b^
Tyrosine	103.2 ± 41.2 ^a^	29.4 ± 12.0 ^b^	36.1 ± 45.5 ^b^
Valine	334.0 ± 27.2 ^a^	358.9 ± 115.8 ^a^	249.5 ± 49.6 ^b^
Methionine	88.5 ± 10.6 ^NS^	100.0 ± 44.1	71.2 ± 25.3
Tryptophane	43.9 ± 7.2 ^NS^	41.0 ± 53.8	59.4 ± 10.0
Phenylalanine	196.8 ± 13.3 ^NS^	242.3 ± 97.3	186.4 ± 46.6
Isoleucine	223.8 ± 15.6 ^ab^	252.2 ± 92.0 ^a^	164.2 ± 36.8 ^b^
Ornithine	98.6 ± 119.1 ^NS^	26.9 ± 443.3	29.4 ± 32.6
Leucine	314.1 ± 14.6 ^NS^	403.5 ± 157.5	301.0 ± 79.9
Lysine	277.3 ± 28.7 ^a^	108.5 ± 82.1 ^b^	134.2 ± 60.0 ^b^
Proline	322.7 ± 56.4 ^b^	520.1 ± 92.3 ^a^	402.4 ± 98.4 ^b^
HICA	30.5 ± 11.01 ^NS^	27.0 ± 14.91	27.1 ± 20.7

Data are expressed as the mean ± SD. Lowercase letters indicate significant differences among groups (*p* < 0.05). ^NS^; Not significant differences among groups.

**Table 6:** Volatile compounds contents of commercial kimchi manufactured in Korea, China, and the United States (μg/g).

	Korea	China	United States
1,2-Epithiopropane	0.03 ± 0.01 ^b^	0.08 ± 0.08 ^a^	0.02 ± 0.021 ^b^
1-Methoxyhexane	-	0.31 ± 0.53	0.21 ± 0.21
1-Pentene-3-ol	0.08 ± 0.03 ^a^	0.01 ± 0.01 ^b^	0.05 ± 0.05 ^ab^
2,3-Butanediol	0.37 ± 0.24 ^NS^	0.25 ± 0.12	0.31 ± 0.26
2-Ethyl-1-hexanol	0.06 ± 0.00 ^NS^	0.10 ± 0.12	0.09 ± 0.03
3-Methyl-1-butanol	0.86 ± 1.1 ^a^	0.38 ± 0.21 ^ab^	0.17 ± 0.18 ^b^
3-Pentenenitrile	0.12 ± 0.03 ^ab^	0.08 ± 0.03 ^b^	0.15 ± 0.09 ^a^
5-Cyano-1-pentene	0.50 ± 0.16 ^b^	0.35 ± 0.20 ^b^	0.96 ± 0.52 ^a^
6-Methyl-5-hepten-2-ol	0.24 ± 0.36 ^NS^	0.04 ± 0.02	0.167 ± 0.09
Acetic acid	1.91 ± 0.40 ^b^	2.79 ± 0.63 ^a^	2.56 ± 0.60 ^a^
Benzeneethanol	0.55 ± 0.25 ^c^	1.50 ± 0.38 ^a^	1.13 ± 0.48 ^b^
Benzenepropanenitrile	3.77 ± 2.20 ^NS^	7.45 ± 5.19	6.77 ± 7.54
cis-3-Hexene-1-ol	0.03 ± 0.01 ^b^	0.05 ± 0.02 ^ab^	0.10 ± 0.11 ^a^
cis-Geraniol	0.27 ± 0.11 ^NS^	0.32 ± 0.09	0.49 ± 0.43
Diallyl disulfide	2.33 ± 1.76 ^a^	0.93 ± 0.86 ^b^	0.92 ± 1.58 ^b^
Diallyl sulfide	0.03 ± 0.01 ^NS^	0.02 ± 0.01	0.02 ± 0.03
Dimethyl trisulfide	0.31 ± 0.2 ^a^	0.24 ± 0.14 ^a^	0.03 ± 0.03 ^b^
endo-Borneol	0.09 ± 0.06 ^b^	0.10 ± 0.07 ^b^	0.21 ± 0.07 ^a^
Ethanol	6.1 ± 6.25 ^NS^	8.23 ± 3.86	5.33 ± 3.13
Ethyl Acetate	0.12 ± 0.11 ^NS^	0.16 ± 0.11	0.16 ± 0.08
Eucalyptol	0.02 ± 0.02 ^b^	0.03 ± 0.02 ^b^	0.08 ± 0.04 ^a^
Eugenol	0.04 ± 0.02 ^b^	0.29 ± 0.08 ^a^	0.09 ± 0.11 ^b^
Linalool	0.10 ± 0.04 ^NS^	0.14 ± 0.06	0.14 ± 0.07
Linalyl_propionate	0.05 ± 0.02 ^b^	0.07 ± 0.03 ^b^	0.15 ± 0.06 ^a^
Methyl allylsulfide	0.09 ± 0.05 ^a^	0.12 ± 0.06 ^a^	0.02 ± 0.02 ^b^
Methyl disulfide	1.14 ± 0.86 ^a^	0.48 ± 0.35 ^b^	0.04 ± 0.03 ^b^
Octanoic acid	0.44 ± 0.26 ^a^	0.11 ± 0.05 ^b^	0.14 ± 0.07 ^b^
S-Methyl thioacetate	0.07 ± 0.08 ^b^	0.43 ± 0.35 ^a^	0.03 ± 0.03 ^b^
Zingiberene	0.33 ± 0.66 ^NS^	0.02 ± 0.02	0.39 ± 0.24
α-Curcumene	0.13 ± 0.15 ^b^	0.05 ± 0.05 ^b^	0.18 ± 0.11 ^a^
β-Bisabolene	0.09 ± 0.09 ^ab^	0.02 ± 0.03 ^b^	0.13 ± 0.09 ^a^
β-Phenethyl acetate	5.05 ± 6.07 ^a^	0.31 ± 0.31 ^b^	0.03 ± 0.02 ^b^
β-Phenethyl_isothiocyanate	0.42 ± 0.24 ^b^	0.60 ± 0.43 ^ab^	0.88 ± 0.59 ^a^
β-Sesquiphellandrene	0.09 ± 0.11 ^ab^	0.08 ± 0.14 ^b^	0.15 ± 0.10 ^a^

Data are expressed as the mean ± SD. Lowercase letters indicate significant differences among groups (*p* < 0.05). ^NS^; No significant differences between groups.

**Table 7:** Antioxidant activities of commercial kimchi manufactured in Korea, China, and the United States.

	Korea	China	United States
TPC (μg/mL)	47.12 ± 2.30 ^a^	38.90 ± 0.98 ^b^	38.52 ± 0.12 ^b^
TFC (μg/mL)	10.26 ± 1.49 ^NS^	9.15 ± 1.79	8.47 ± 2.16
DPPH (%)	44.34 ± 1.28 ^a^	41.87 ± 1.82 ^a^	34.34 ± 1.45 ^b^
TAC (μM)	3556.44 ± 113.08 ^a^	2734.67 ± 87.93 ^b^	2816 ± 37.46 ^b^
FRAP (μM)	1956.56 ± 19.05 ^a^	1398.95 ± 5.16 ^b^	1414.32 ±32.59 ^b^

Data are expressed as the mean ± SD. Lowercase letters indicate significant differences betwen groups (*p* < 0.05). ^NS^; No significant differences among groups. TPC; total phenol content, TFC; total flavonoid content, DPPH; 2,2-diphenyl-1-picrylhydrazyl, TAC; total antioxidant capacity, FRAP; ferric reducing antioxidant power.

## Data Availability

The data presented in this study are available in the article.

## Supplementary Materials

The following are available online at https://www.mdpi.com/article/10.3390/foods10102488/s1, Table S1: Information on commercial kimchi manufactured in Korea, China, and United States.
