# Supplementary material for: Comparison of Quality Characteristics of Commercial Kimchi Manufactured in Korea, China, and the United States
Source: Foods. 2021 Oct 18;10(10):2488. doi: 10.3390/foods10102488 (PMC8535366; doi:10.3390/foods10102488)
Supplement: Supplementary file 1 [file foods-10-02488-s001.zip › foods-1408164-supplementary.pdf]

**Supplementary Table S1.** Information on commercial kimchi manufactured in Korea, China, and United States.

| Country | Company (Product name) | Main Ingredients |
|---|---|---|
| Korea (K-1) | CJ (Sliced cabbage kimchi) | Cabbage, radish, red pepper powder, CJ anchovy sauce, CJ paste, CJ salted shrimp, salt, sugar, onion, green onion, garlic, ginger, probiotic broth, fructose |
| Korea (K-2) | Our home (Sliced cabbage kimchi) | Cabbage, radish, kimchi seasoning sauce, pepper powder, anchovy sauce, salted shrimp, garlic, ginger, salt, sugar, onion, green onion, probiotic broth, fructose, MSG |
| Korea (K-3) | Daesang (Sliced cabbage kimchi) | Cabbage, radish, glutinous rice paste, onion, red pepper powder, green onion, anchovy sauce, sugar, garlic, ginger, probiotic, salt |
| Korea (K-4) | Pulmuone (Sliced cabbage kimchi) | Cabbage, radish, glutinous rice paste, onion, red pepper powder, green onion, anchovy sauce, sugar, garlic, ginger, leek, probiotic, salt |
| Korea (K-5) | Dongwon (Sliced cabbage kimchi) | Cabbage, radish, glutinous rice paste, onion, red pepper powder, green onion, anchovy sauce, sugar, garlic, ginger, leek, pear, persimmon, kelp broth, salt, probiotic broth |
| China (C-1) | Daesang (Sliced cabbage kimchi) | Cabbage, radish, red pepper powder, green onion, onion, sugar, garlic, ginger, anchovy sauce, MSG, sorbitol, xanthan gum, corn syrup, leek, probiotic |
| China (C-2) | Pulmuone (Sliced cabbage kimchi) | Cabbage, radish, anchovy sauce, salt, red pepper powder, green onion, onion, sugar, garlic, ginger, xanthan gum, corn syrup, glutinous rice paste, probiotic |
| China (C-3) | NISSI FOOD (Kimchi) | Cabbage, radish, onion, anchovy sauce, red pepper powder, salt, ginger, malt sugar, xanthan gum |
| China (C-4) | Gyeongbokgung (Kimchi) | Cabbage, radish, anchovy sauce, onion, green onion, red pepper powder, garlic, ginger, alcohol, citric acid, xanthan gum, food color(turmeric), glutinous rice paste |
| China (C-5) | Sankou (Kimchi) | Cabbage, radish, anchovy sauce, salted shrimp, salt, sugar, onion, green onion, red pepper powder, garlic, ginger, glutinous rice paste, apple puree, kelp, potassium sorbate |
| United States (U-1) | Mother in law (House kimchi) | Cabbage, onion, red pepper powder, salt, garlic, ginger, organic sugar, anchovy sauce |
| United States (U-2) | Seoul (Original kimchi) | Cabbage, red pepper powder, carrot, garlic, green onion, cane sugar, sesame, onion, sea salt, ginger |
| United States (U-3) | Wildbrine (Kimchi) | Cabbage, green onion, sea vegetable, red pepper powder, sea salt, garlic, ginger |
| United States (U-4) | COSMOS (Kimchi) | Cabbage, kale, water, carrot, sugar, red pepper powder, garlic, sea salt, ginger, onion |
| United States (U-5) | Mother in law (Vegan) | Cabbage, onion, welsh onion, red pepper powder, salt, garlic, ginger, organic sugar |
| United States (U-6) | Seoul (Vegan kimchi) | Cabbage, red pepper powder, carrot, garlic, cane sugar, welch onion, sea salt, ginger |
